# Bioactive Natural Products for Chemical Control of Microorganisms: Scientific Prospecting (2001–2021) and Systematic Review

**DOI:** 10.3390/molecules27185917

**Published:** 2022-09-12

**Authors:** Bruno Fonsêca Feitosa, Charlene Maria de Alcântara, Amanda Beatriz Sales de Lima, Adriano Sant’Ana Silva, Alfredina dos Santos Araújo, Mônica Tejo Cavalcanti, Edna Mori, Isaac Moura Araújo, Pablo Antonio Maia de Farias, Polrat Wilairatana, Henrique Douglas Melo Coutinho

**Affiliations:** 1 Faculty of Food Engineering, State University of Campinas, Monteiro Lobato, 80, University City “Zeferino Vaz”, Campinas 13083-862, SP, Brazil; 2 Academic Unit of Food Technology, Federal University of Campina Grande, Jairo Vieira Feitosa, 1770, Pombal 58840-000, Pereiros, PB, Brazil; 3 Department of Rural and Animal Technology, State University of Southwest Bahia, Praça Primavera, 40, Itapetinga 45700-000, BA, Brazil; 4 National Institute of the Semiarid Region, Francisco Lopes de Almeida, Serrotão, Campina Grande 58434-700, PB, Brazil; 5 CECAPE College. Av. Padre Cícero, 3917, Juazeiro do Norte 63024-015, CE, Brazil; 6 Department of Biological Chemistry, Regional University of Cariri, Av. Cel. Antonio Luiz, 1161, Crato 63105-000, CE, Brazil; 7 Department of Clinical Tropical Medicine, Faculty of Tropical Medicine, Mahidol University, Bangkok 10400, Thailand

**Keywords:** anti-bacterial, bioactive compounds, essential oils, extracts, scientific articles

## Abstract

The inappropriate use of synthetic antibiotics has become a global public health problem. Therefore, the study of new alternatives for the treatment of infectious diseases is relevant and natural bioactive products are on the rise. This study conducted a scientific prospection of bioactive natural products with promising applications in the chemical control of microorganisms. A systematic review of the most recent articles was performed according to the following three steps: (i) eligibility assessment, (ii) screening, and (iii) inclusion of articles and information extraction. There has been an increase in the number of scientific publications on bioactive natural products for microbial control in the CAPES and SciELO databases (2001–2021). Seventeen relevant articles were included, most of which focused on extracts. Ascorbic acid, chlorogenic acid, chrysin, and quercetin were the most cited compounds. Natural products were shown to be effective in inhibiting more than 30 microorganisms. A discussion was presented on the research trends.

## 1. Introduction

Injudicious use of conventional synthetic antibiotics in the treatment of infectious diseases has become a global public health problem. Pathogenic microorganisms evolve by developing adaptation and resistance mechanisms, such as production of inactivating enzymes, decreased membrane permeability, and efflux pumps for antibiotics. Multidrug resistance has been reported in several clinically relevant bacteria, including *Escherichia coli*, *Klebsiella pneumoniae*, *Pseudomonas aeruginosa*, and *Staphylococcus aureus* [1,2,3].

The World Health Organization (WHO) monitors global trends in evidence-based healthcare and has supported the implementation (2014–2023) of traditional and complementary medicine strategies. Traditional and complementary medicine is an important health resource for modern therapy and in it might lie important solutions to the development and evolution of multidrug-resistant strains [4,5]. For instance, Brazil has a great diversity of medicinal plants widely investigated for scientific and technological applications and intensively used as herbal medicines [6,7].

Several chemical compounds can be produced and extracted from a single species [8,9]. Medicinal plants can be used to produce standardized extracts, essential oils, and purified phytochemicals with biological activities [10,11]. Bioactive natural products, which can be used separately or in combination, induce changes in membrane permeability or damage proteins and nucleic acids in microbial cells [12]. Studies have demonstrated that several bioactive natural products show effective antagonistic activity against foodborne pathogens and spoilage microorganisms [13,14].

This study aimed to conduct a scientific prospection and systematic review of promising bioactive natural products for chemical control of microorganisms. Two databases were selected and strategies were applied for the search, collection, and treatment of prospection data (2001–2021). Protocols were developed for the selection of the most recent articles and data extraction, comprising the following three steps: (i) eligibility assessment, (ii) screening, and (iii) article inclusion and information extraction. A discussion was presented on the main sources of extraction, importance of natural bioactive products, most cited bioactive natural compounds, microorganisms and inhibition effectiveness, and perspectives on research trends.

## 2. Results and Discussion

### 2.1. Scientific Prospecting of Data

The results of the searches carried out in CAPES and SciELO databases are presented in Table S1 (Supplementary Materials). The keywords with the highest quantitative contribution were (“essential oil” OR extract) AND antib* for the CAPES database and (“essential oil” OR extract) AND antimicr* for the SciELO database, both in English. Search strategies containing English keywords retrieved a larger number of articles, given the international nature of this language. From a total of 266,936 scientific articles retrieved, 256,780 were published in peer-reviewed journals indexed in the CAPES database. The CAPES database had a higher number of published articles in both languages, as expected, because this database also includes publications indexed by SciELO. Sample representativeness was the lowest when the keyword “fitoquímico” (phytochemical) was used as a descriptor in term 1, with a reduction in the number of retrieved papers according to the following order of descriptors in term 2: antimicr* > antib* > bactericidal > biocidal > “chemical agent”.

### 2.2. Annual Evolution of Studies (2001–2021)

**Figure 1:**
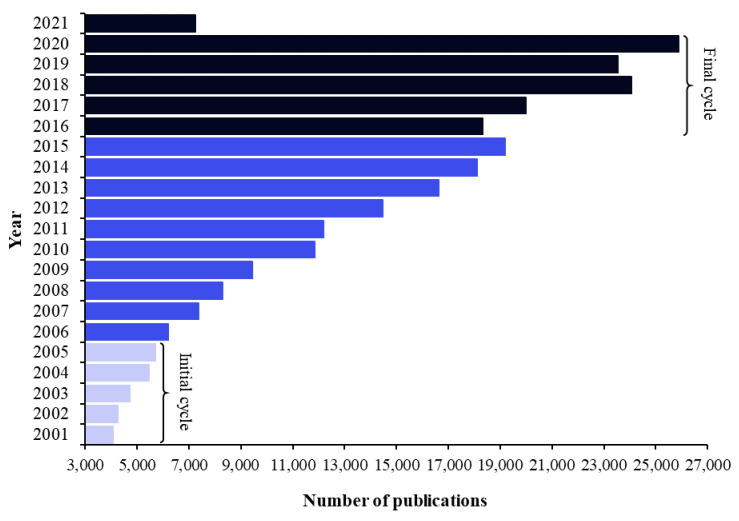
Annual number of scientific publications identified in the CAPES database. The search strategy was (“essential oil” OR extract) AND antib*.

The annual distribution (2001–2021) of articles retrieved from the CAPES database by using the keywords (“essential oil” OR extract) AND antib* is depicted in Figure 1. Initial and final cycles were defined as the first and last 5 completed years, respectively, of the study temporal range (21 years).

The number of publications in the CAPES database increased from 2001 to 2021. An increase in the number of studies in relation to the previous number occurred every year, except in 2016 (*n* = 18,329) and 2019 (*n* = 23,539). More than 20,000 articles were published in the last 5 completed years (final cycle). This number was at least four times higher than that in the initial cycle (2001–2005). It was estimated that more articles will be published in subsequent years than in previous years, given that, up to the first quarter of 2021, 7234 studies had been published. Thus, >26,000 articles on the use of bioactive natural products for microbial control are expected to be published in 2021. WHO’s incentive for countries to generate evidence-based policies and strategic plans for the use of medicinal plants might be related to the increase in research in recent years [4].

**Figure 2:**
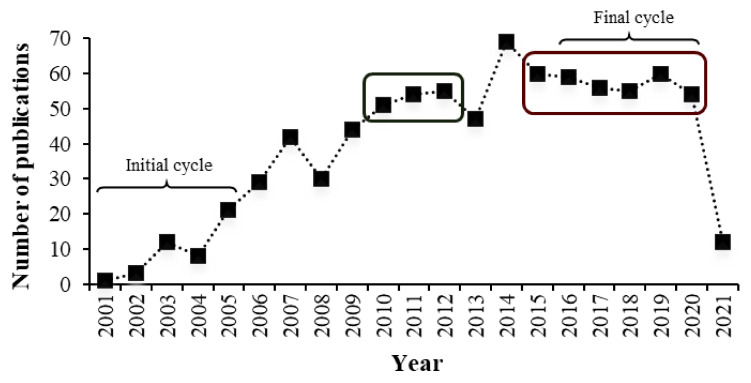
Annual number of scientific publications identified in the SciELO database. The search strategy was (“essential oil” OR extract) AND antimicr*.

The annual distribution (2001–2021) of scientific articles indexed in the SciELO database, identified by the keywords (“essential oil” OR extract) AND antimicr*, is depicted in Figure 2.

The number of publications in the SciELO database also increased over the past 21 years. In 2004, 2008, 2013, and 2015, there was a low number of publications compared with previous years. From 2015 to 2020, the number of publications remained constant (≈57), similar to that observed from 2010 to 2012 (≈53). The highest peak occurred in 2014, with 69 scientific articles. The final cycle (last 5 completed years) had about five times the number of publications than the initial cycle. It was estimated that the number of publications in 2021 would not surpass that of 2020, as only 12 articles had been published in the first quarter. The national scenario of disruptions and limitations imposed by the SARS-CoV-2/COVID-2019 pandemic might have hindered the annual evolution of experimental studies.

### 2.3. Ranking of Thematic Areas and Journals

Ranking of the thematic areas and journals according to the number of scientific articles published in CAPES and SciELO databases is presented in Table 1.

The thematic areas Chemistry (23.52%, *n* = 35,797), Engineering (16.71%, *n* = 25,428), and Bacteria (15.98%, *n* = 24,329) had the highest number of publications in the CAPES database. More specific areas, such as antibacterial agents, antibacterial activity, and antimicrobial agents, were also identified. The microorganisms *E. coli* and *S. aureus* ranked 5th and 10th, respectively, as thematic areas, suggesting that they are the major species investigated in inhibition tests with bioactive natural products. The journal *Molecules* had more than 5000 publications, representing about 70% of the total number of articles (*n* = 7846). The other journals with the highest number of publications on the subject were Journal of Applied Microbiology (6.93%, *n* = 517), Journal of Essential Oil-Bearing Plants (4.86%, *n* = 363), and Industrial Crops and Products (4.41%, *n* = 329).

Articles retrieved from SciELO were classified into the major thematic areas Agricultural Sciences (29.48%, *n* = 278), Health Sciences (26.30%, *n* = 248), and Biological Sciences (25.03%, *n* = 236). These thematic areas were comprehensive, with the global field of sciences accounting for over 800 publications. Articles can be classified into more than one area of publication or theme, generating duplicity of citations and variation in the number of themes. Of the scientific journals indexed in the SciELO database, Revista Brasileira de Pharmacognosia was the one with the highest number of publications on the subject (25.97%, *n* = 100), in agreement with the thematic area ranking 2nd (health sciences). The three journals with the largest numbers of scientific articles were discontinued from the SciELO database, namely Revista Brasileira de Pharmacognosia, Revista Brasileira de Plantas Medicinais, and Brazilian Journal of Microbiology. The scopes of the ranked journals are in accordance with the main thematic areas. All journals reported the impact factor, as identified in the 2019 Journal Citation Reports, with the exception of Revista Brasileira de Plantas Medicinais. Nevertheless, the impact factor was not directly proportional to the number of published articles.

### 2.4. Country of Origin of Journals

**Figure 3:**
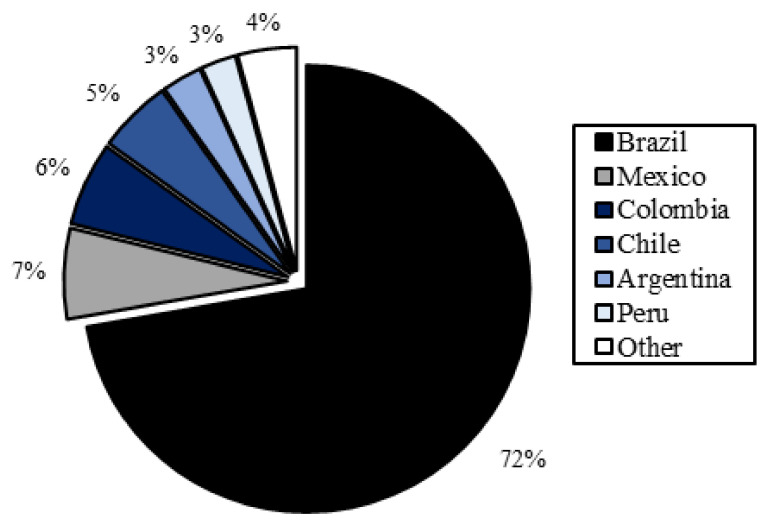
Countries with the highest proportion of scientific publications on bioactive natural products for microbial control in the SciELO database. The search strategy was (“essential oil” OR extract) AND antimicr*.

Figure 3 shows the number of publications retrieved from the SciELO database according to the country of origin of scientific journals.

A total of 6 countries had the highest number of publications, totaling 822 articles. Brazil accounted for 72% (*n* = 594) of the publications, which was expected, given that the SciELO database comprises a wide range of Brazilian scientific journals. Mexico, Colombia, Chile, Argentina, and Peru were the following major publishing countries, demonstrating the importance of research on bioactive natural products for microbial control in these countries. Only 4% (*n* = 35) of publications stemmed from other countries.

### 2.5. Relevant Recent Research

Initially, 822 records were identified using the advanced search table. In the first stage of selection, 66 articles were pre-selected, 22 of which were excluded. In the second stage (full-text reading), 27 articles were excluded. In the third stage, 17 relevant articles were selected. The information of interest was extracted, grouped, and presented in Table 2 and Figure S1 (Supplementary Materials).

### 2.6. Main Sources of Extraction

Bioactive natural products of plant origin predominated in the retrieved studies. Only two articles used an extraction source of animal origin (propolis) [25,27]. The other 15 studies investigated different botanical species, suggesting interest of the scientific community in identifying new potential bioproducts from a diversity of plant species. Most studies extracted compounds from leaves [5,16,17,18,19,20,21,28,29,30], followed by peels [15,17,29], aerial parts [22,23,26], stem [17,18], flower [18], and roots [24]. According to Yuan et al. [31], leaves provide higher essential oil yields than other plant parts.

### 2.7. Importance of Natural Bioactive Products

Extracts and essential oils were the most frequent keywords (Table S1, Supplementary Materials). Studies that used extracts predominated [15,17,18,19,20,21,22,24,25,26,27,29,30]. Plant extracts can be obtained by processes that concentrate the desired material into a matrix of greater chemical complexity [32]. The low extraction yield (<1%) of essential oils may be a limiting factor, as argued by Wajs-Bonikowska et al. [33]. Ethanol and water were the major solvents for obtaining bioactive natural products. Hydrodistillation was mainly used for essential oil extraction [5,16,23,28] and maceration for extract preparation [17,20,22,24,25,26,29,30].

### 2.8. Most Cited Bioactive Natural Compounds

Bioactive products were categorized into classes, subclasses, and specific compounds. A higher frequency of flavonoids and terpenoids was observed. Sesquiterpenes and caryophyllenes were the most frequently identified subclasses of terpenes. The 3D structure and characteristics of the most cited compounds are shown in Table 3. Ascorbic acid, chlorogenic acid, chrysin, and quercetin were associated with various bioactive properties, such as antioxidant, anti-inflammatory, antiviral, antiallergic, hepatoprotective, neuroprotective, antiasthmatic, antidiabetic, and antidepressant properties [34,35,36,37]. It is important to note that the identification of some constituents may be limited by the analytical method used. Some identification techniques depend on analytical standards and may not be able to screen all major compounds of extracts and essential oils. Therefore, quantified constituents may or may not be the most important compounds in terms of composition and bioactivity.

### 2.9. Microorganisms and Inhibition Effectiveness

Inhibition effectiveness was tested against more than 30 microorganisms. Cunha et al. [20] found that the foliar extract of *Senna rugosa* was not effective in inhibiting *Aspergillus niger* ATCC 10535, *Candida albicans* ATCC 90028, *Klebsiella oxytoca* ATCC 49131, *Penicillium expansum* ATCC 1117, or *Salmonella* Typhimurium ATCC 14028. The other relevant articles reported partial or total efficacy of bioactive natural products, demonstrating the high potential of biocompounds for the control of microorganisms. Efficacy in inhibition tests is directly associated with biocompound concentration, treatment time, and microbial resistance. Various studies have carried out tests against multi-drug resistant strains, such as *S. aureus* (Gram-positive) and *E. coli* (Gram-negative), explaining the high rank of these thematic areas with regard to the number of published studies (Table 1). *C. albicans* was the most studied fungus [15,17,19,20,21,26,27].

### 2.10. Perspectives on Research Trends

Relevant articles demonstrated the promising action of bioactive natural products in microbial control. The following aspects should be considered and further explored:

1. Inclusion of extract or essential oil in food matrices may contribute to the bioactivity, bioavailability, and bioaccessibility of bioactive compounds of interest; compounds can be introduced into matrices via alternative techniques, such as marinating, sprinkling, soaking, and brushing.
2. In vitro and in vivo results are important to substantiate claims of the functional properties of bioactive ingredients in foods, contributing to healthiness and ensuring consumers’ rights to information.
3. Extraction of bioactive compounds and development of bioactive natural products from food industry by-products has high scientific, technological, socioeconomic, and environmental appeal; these materials can also be used in active and intelligent packaging.
4. Reports of the direct application of purified phytochemicals and bioactive natural dyes/pigments for food enrichment are still scarce; viable systems may be developed to strengthen the metabolic activity of beneficial microorganisms, whether added, natural, or autochthonous (acid-lactic bacteria and/or yeasts, probiotic or not).
5. Compound protection technologies, such as microencapsulation, nanoencapsulation, and nanoemulsion, can optimize bioactivity, bioavailability, and bioaccessibility, thereby enhancing product stability and ease of transport, storage, and distribution.

## 3. Material and Methods

**Figure 4:**
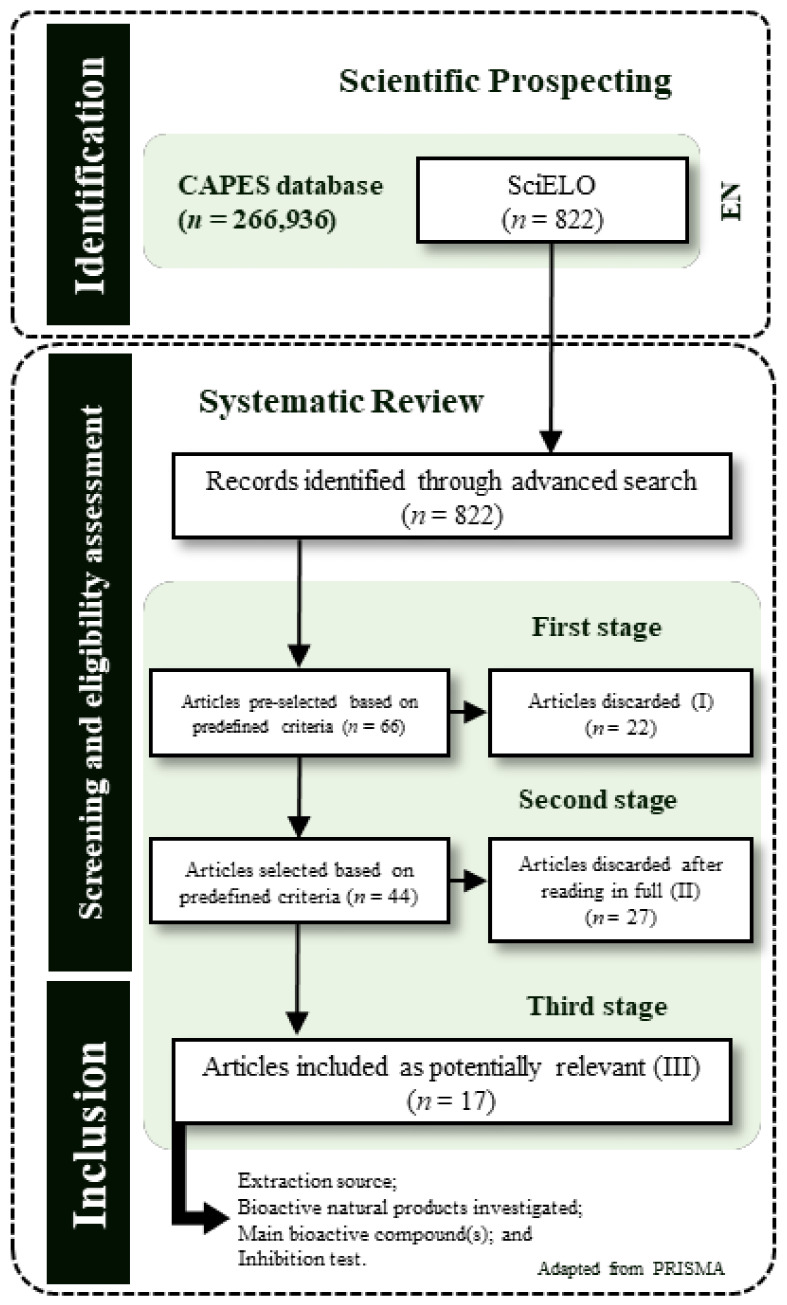
Flowchart of the mixed method used to search the literature for relevant articles on bioactive natural products for microbial control. En, articles written in English.

This study used a mixed method, consisting of scientific prospecting followed by a systematic review of the literature (Figure 4) based on the Preferred Reporting Items for Systematic Reviews and Meta-Analyses (PRISMA) [38], including procedures adapted from the work of Melo et al. [39] and Bezerra and Pinheiro [40].

### 3.1. Scientific Prospecting of Data

#### 3.1.1. Sources of Information

The following two electronic databases were searched: the CAPES database (*Portal de Periódicos CAPES* of the Brazilian Federal Agency for Support and Evaluation of Graduate Education), which provides democratic and easy access to several databases of high scientific relevance [41], and the Scientific Electronic Library Online (SciELO) database, which is the main cooperative electronic portal for open-access scientific journals in Latin America [42]. No contact was made with the authors to identify additional studies.

#### 3.1.2. Search Strategies

Searches for articles on bioactive natural products for microbial control, written in Portuguese or English, were carried out in the advanced search tab of each database. Truncated terms, quotation marks, and Boolean operators were used to construct the search strategy, according to the particularities, supported characters, and instructions provided by the databases. Conference proceedings, technical books, monographs, dissertations, and theses were excluded by applying specific filters. Searches were performed between 27 April and 6 May 2021. Time of publication was restricted to the past 21 years. The combination of keywords and descriptors, presented in detail in Table S2 (Supplementary Materials), can be summarized as follows: bioactiv* AND (antib*; antimicr*; bactericidal; biocidal; “chemical agent”), phenol* AND (antib*; antimicr*; bactericidal; biocidal; “chemical agent”), phytochemical AND (antib*; antimicr*; bactericidal; biocidal; “chemical agent”); and “essential oil” OR extract AND (antib*; antimicr*; bactericidal; biocidal; “chemical agent”).

#### 3.1.3. Data Processing

The keyword and language with the highest quantitative contributions were used for data processing. The data were tabulated and analyzed in terms of the following quantitative variables: annual number of publications (2001–2021), ranking of thematic areas, journals with the highest number of articles published, and country of origin of journals (SciELO database only). The results are presented in tables or figures, constructed using Microsoft^®^ Excel 2013.

### 3.2. Systematic Review Protocol

Publications were identified and selected using the keyword and language with the highest quantitative contribution in the SciELO database. A protocol was used to select the most recent articles and extract the data, consisting of the following three steps: (i) eligibility assessment, (ii) screening, and (iii) article inclusion and extraction of information. The risk of bias was not assessed. Eligibility was defined as meeting the totality of the following criteria (yes = 100%):

1. Was the scientific paper published between 2020 and the current date (2021)?
2. Can the overall objective of the research be easily identified?
3. Is the source of bioactive natural products specified?
4. Was the bioactive natural product used in the form of an extract or essential oil?
5. Is the preparation procedure described clearly and in detail?
6. Has at least one compound responsible for biological activity (rather than a group or class) been identified using adequate analytical techniques?
7. Were inhibition tests performed against at least one specific microorganism?
8. Is the analytical method or technique described clearly and in detail?
9. Is the effectiveness of microbial inhibition reported?
10. Are the results and/or conclusions aligned with the overall objectives of the study?

In the first step, articles that did not meet the eligibility criteria were excluded. In the second step, articles were screened by two reviewers, who read the texts in full in a double-blind system and selected relevant articles. Disagreements were discussed until consensus was reached and the opinion of a third reviewer was requested for final decision making. In the third step, articles were included on the basis of shared decisions, and the following information was extracted: natural source of bioactive products (species and organ), type of bioactive natural product (extract or essential oil, solvent, and extraction method), main bioactive compounds and major components of the natural product, and inhibition test (microorganism species and efficacy assessment).

Efficacy was categorized as yes, partial, or no. Compounds were classified as effective (yes) when they achieved complete efficacy at a given concentration and/or during a given incubation time; as partial when they showed limited efficacy against at least one type of microorganism, regardless of the concentration and/or incubation time tested; and as ineffective (no) when they did not achieve significant results at any concentration or incubation time against any microorganism. Extracted data were organized in tables and/or figures and analyzed in terms of main sources of extraction, importance of natural bioactive products, most cited bioactive natural compounds, microorganisms and inhibition effectiveness, and perspectives on research trends.

## 4. Conclusions

The research trends indicated that more than 26,000 articles on the potential of bioactive natural products for chemical control of microorganisms will be published in the coming years in journals indexed by CAPES. *E. coli* and *S. aureus* were the main species investigated in inhibition tests with bioactive natural products. In the SciELO database, more than 800 articles categorized into the field of Sciences were identified, and the *Revista Brasileira de Pharmacognosia* was the major publishing journal, with 100 publications (25.97%). Scientific interest in the subject has increased in Brazil and in countries such as Mexico, Colombia, Chile, Argentina, and Peru.

The major extraction sources were of plant origin, mainly leaves, peels, and aerial parts. The importance of extracts and essential oils was evidenced. These materials were obtained mainly by maceration and hydrodistillation, respectively, with ethanol and water as solvents. Ascorbic acid, chlorogenic acid, chrysin, and quercetin were the most cited bioactive compounds. Partial and/or total inhibition of at least 30 different microorganisms by bioactive natural products was identified, being related to compound concentration, treatment time, and microbial resistance. Relevant articles demonstrated the promising action of bioactive natural products in the chemical control of microorganisms. Future trends were discussed.

## Figures and Tables

**Table 1:** Ranking of thematic fields and journals with the highest number of publications on bioactive natural products for microbial control.

Ranking	Thematic Field	Number of Publications	%	Journal	Number of Publications	%	Impact Factor ^1^
CAPES database ^2^							
1	Chemistry	35,797	23.52	*Molecules*	5222	69.97	3.267
2	Engineering	25,428	16.71	*Journal of Applied Microbiology*	517	6.93	3.066
3	Bacteria	24,329	15.98	*Journal of Essential Oil-Bearing Plants*	363	4.86	0.824
4	Antioxidants	14,472	9.51	*Industrial Crops and Products*	329	4.41	4.244
5	*Escherichia coli*	13,665	8.98	*Food Chemistry*	237	3.18	6.306
6	Antibacterial agents	10,113	6.64	*Journal of Agricultural and Food Chemistry*	216	2.89	4.192
7	Agriculture	9510	6.25	*Microbial Pathogenesis*	159	2.13	2.914
8	Antibacterial activity	6906	4.54	*Journal of Food Science*	152	2.04	2.478
9	Antimicrobial agents	6415	4.21	*Food Control*	151	2.02	4.258
10	*Staphylococcus aureus*	5570	3.66	*Chemistry & Biodiversity*	117	1.57	2.039
Total		152,205	100.00		7846	100.00	
SciELO database ^3^							
1	Agricultural Sciences	278	29.48	*Revista Brasileira de Farmacognosia* ^4^	100	25.97	1.407
2	Health Sciences	248	26.30	*Revista Brasileira de Plantas Medicinais* ^4^	67	17.40	ND
3	Biological Sciences	236	25.03	*Brazilian Journal of Microbiology* ^4^	49	12.73	2.428
4	Multidisciplinary	95	10.07	*Ciência Rural*	33	8.57	0.556
5	Exact and Earth Sciences	64	6.79	*Food Science and Technology*	31	8.05	1.637
6	Engineering	22	2.33	*Anais da Academia Brasileira de Ciências*	26	6.75	1.280
7				*Brazilian Archives of Biology and Technology*	26	6.75	0.579
8				*Química Nova*	19	4.94	0.668
9				*Brazilian Journal of Pharmaceutical Sciences*	17	4.42	0.814
10				*Journal of the Brazilian Chemical Society*	17	4.42	1.399
Total		943	100.00		385	100.00	

^1^ 2019 Journal Citation Reports. ^2^ Searched for (“essential oil” OR extract) AND antib*. ^3^ Searched for (“essential oil” OR extract) AND antimicr*. ^4^ Journals removed from SciELO. ND, not identified.

**Table 2:** Information extracted from relevant articles on bioactive natural products for microbial control.

No.	Natural Source		Bioactive Natural Product			Bioactive Components			Inhibition Tests		Reference
	Species	Organ	E/EO	Solvent	Extraction Method	Class	Subclass	Compound	Microorganism	Efficacy (Y/P/N)	
1	*Persea americana* ‘Hass’ *Cocos nucifera* L.	Peel	E	Water Ethanol	Ultrasonication	-	-	Ascorbic acid Caffeic acid Gallic acid Oxalic acid Catechin Epicatechin Procyanidin B1 Procyanidin B2	*Candida albicans* *Shigella dysenteriae* *Staphylococcus aureus*	Y	[15]
2	*Schinus molle* L.	Leaves	EO	Water	Hydrodistillation	-	Monoterpene Sesquiterpene	-	*Corynebacterium* spp. *Pseudomonas* spp. *Staphylococcus* spp. *Streptococcus* spp.	Y	[16]
3	*Albizia inundata* (Mart.) Barneby & J.W.Grimes	Stems Bark Leaves	E	Petroleum ether Hexane Methanol	Maceration	-	-	Lupenone	*Bacillus subtilis* ATCC 6633 *C. albicans* ATCC 18804 *E. coli* ATCC 94863 *Micrococcus luteus* ATCC 10240 *P. aeruginosa* ATCC 14028 *Salmonella choleraesuis* ATCC 14028 *S. aureus* ATCC 6538	P	[17]
4	*Lantana camara* L. *Lippia dulcis* T. *Petiveria alliacea* L.	Stems Flowers Leaves	E	Ethanol	Percolation	-	Anthraquinone Coumarin	7-Hydroxycoumarin β-Sitosterol Quercetin	*E. coli* ATCC 25922 *Proteus vulgaris* ATCC 6380 *P. aeruginosa* ATCC 9027 *S. aureus* ATCC 25923	P	[18]
5	*Talinum paniculatum* (Jacq.) Gaertner	Leaves	E	Water Ethanol Fractions: Ethyl acetate Hexane	Percolation	-	-	Ascorbic acid Benzoic acid Caffeic acid Chlorogenic acid Ferulic acid Campesterol Sitosterol Stigmasterol	*C. albicans* ATCC 10231 *C. albicans* ATCC 90028	P	[19]
6	*Senna rugosa*	Leaves	E	Ethanol Fractions: Ethyl acetate and n-Hexane	Maceration	Flavonoid Tannin	Anthraquinone Triterpene	Rutin	*Candida famata* ATCC 62894 *Candida* Krusei ATCC 34135 *Candida tropicalis* ATCC 28707 *P. aeruginosa* ATCC 27853 *S. aureus* ATCC 29313	Y	[20]
									*Aspergillus niger* ATCC 10535 *C. albicans* ATCC 90028 *Klebsiella oxytoca* ATCC 49131 *Penicillium expansum* ATCC 1117 *Salmonella* Typhimurium ATCC 14028	N	
7	*Eugenia uniflora*	Leaves	E	Ethanol	Percolation	Flavonoid	-	Quercetin	*C. albicans* *K. oxytoca* *S. aureus*	Y	[21]
8	*Salix babylonica*	Aerial parts	E	Methanol	Maceration	Steroid Quinone Saponin	Coumarin Flavonol Phlorotannin Lactone Triterpene	Carvacrol Limonene Terpinene Thymol	*B subtilis* 6633 *E. coli* 35218 *Listeria monocytogenes* 19113 *P. aeruginosa* 9027 *Salmonella typhi* 14028 *S. choleraesuis* 10708 *S. aureus* ATCC 6538	P	[22]
9	*Vernonia chalybaea*	Aerial parts	EO	Water	Hydrodistillation	-	-	β-Caryophyllene β-Elemene Bicyclogermacrene Caryophyllene oxide	*Candida* spp. *Trichophyton rubrum*	Y	[23]
10	*Jatropha dioica* Seseé	Roots	E	Methanol Fractions: Ethyl acetate n-Hexane Water	Maceration	-	Diterpene	Citlalitrione	*Clavibacter michiganensis* subsp. *michiganensis* *Pseudomonas syringe* pv. *tomato* *Xanthomonas campestris* pv. *vesicatoria*	Y	[24]
11	Propolis		E	Ethanol	Maceration	Flavonoid	-	Chrysin Galangin Naringenin Pinocembrin Quercetin	*E. coli* ATCC 25922 *Listeria innocua* *S. Typhimurium* ATCC 14028 *S. aureus* ATCC 29213B	S	[25]
12	*Helichrysum plicatum* subsp. *plicatum*	Aerial parts	E	Methanol	Maceration Soxhlet extraction	-	Luteolin	Chlorogenic acid Dicaffeoylquinic acid Isoquercitrin Luteolin-7-O-glycoside Naringenin-O-hexoside	*C. albicans* ATCC 10231 *E. coli* ATCC 8739 MRSA *K. pneumoniae* ATCC 4352 *Proteus mirabilis* ATCC 14153 *P. aeruginosa* ATCC 27853 *S. aureus* ATCC 6538 *Staphylococcus epidermidis* ATCC 12228	P	[26]
13	Propolis		E	Water Dimethyl sulfoxide Ethanol Propylene glycol	Ultrasonication Percolation	-	-	Ellagic acid Chrysin Myricetin Quercetin	*Aspergillus flavus* ATCC 15517 *A. niger* ATCC 9642 *C. albicans* ATCC 10251 *E. coli* 0157H7 ATCC 43888 *L. monocytogenes* ATCC 13932 *S. Typhimurium* ATCC14028 *S. aureus* ATCC 29213 *Streptococcus mutans* UA159 ATCC 700610 *Penicillium carneum* IBT 14042	S	[27]
14	*Bixa orellana* Labil	Leaves	EO	Water	Hydrodistillation	-	-	1R-α-Pinene α-Guayene β-Bisabolene β-Farnesene β-Pinene Caryophyllene	*E. coli* ATCC 25922 *S. aureus* ATCC 25923	P	[28]
15	*Jatropha platyphyll*	Peel Leaves	E	Methanol	Maceration	Alkaloid Flavonoid Saponin Tannin Terpene	-	-	*Aspergillus parasiticus*	Y	[29]
16	*Campomanesia aurea*	Leaves	EO	Water	Hydrodistillation	-	Monoterpene Sesquiterpene	-	*L. monocytogenes* ATCC 13932 *L. monocytogenes* ATCC 19114 *L. monocytogenes* ATCC 7644	Y	[5]
17	*Origanum vulgare*	Leaves	E	Ethanol	Cold maceration	Sterol Flavonoid Tannin	Coumarin Flavanone Flavonol Lactone Sesquiterpene Triterpene	-	*S. mutans* *Streptococcus sobrinus* ATCC	Y	[30]

MRSA, methicillin-resistant *Staphylococcus aureus*; E, extract; EO, essential oil; Y, yes; P, partial; N, no; ATCC, American Type Culture Collection.

**Table 3:** Bioactive compounds identified in articles included in this review.

No.	Compound	3D Structure	Characteristics	References
1	Ascorbic acid	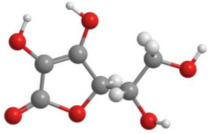	Formula: C_6_H_8_O_6_ MW: 176.12 g mol^−1^ Density: 1.65 g cm^−3^ BP: 553.00 °C	[15,19]
2	Chlorogenic acid	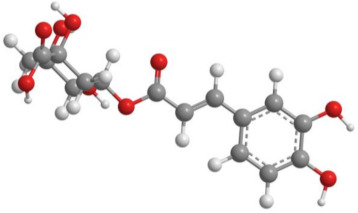	Formula: C_16_H_18_O_9_ MW: 354.31 g mol^−1^ Density: 1.28 g cm^−3^ BP: 665.00 °C	[19,26]
3	Chrysin	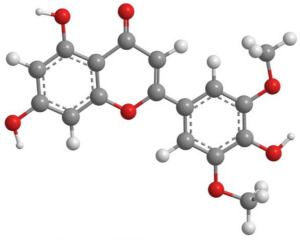	Formula: C_15_H_10_O_4_ MW: 254.22 g mol^−1^ Density: 1.27−1.40 g cm^−3^ BP: 285.50 °C	[25,27]
4	Quercetin	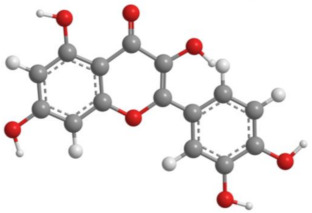	Formula: C_15_H_10_O_7_ MW: 302.20 g mol^−1^ Density: 1.80 g cm^−3^ BP: 316.00–317.00 °C	[18,21,25,27]

MW, molecular weight; BP, boiling point.

## Supplementary Materials

The following are available online at https://www.mdpi.com/article/10.3390/molecules27185917/s1, Table S1. Results of the keyword searches for articles in Portuguese (PT) and English (EN); Table S2. List of keywords used to search for scientific articles on bioactive natural products for 15 microbial control in Portuguese and English. Figure S1. Graphical abstract of scientific information on bioactive natural products for microbial control.
