# Supplementary material for: Bioactive Natural Products for Chemical Control of Microorganisms: Scientific Prospecting (2001–2021) and Systematic Review
Source: Molecules. 2022 Sep 12;27(18):5917. doi: 10.3390/molecules27185917 (PMC9505009; doi:10.3390/molecules27185917)
Supplement: Supplementary file 1 [file molecules-27-05917-s001.zip › molecules-1899740-supplementary.pdf]

molecules

Supplementary Materials

Bioactive Natural Products for Chemical Control of Microorganisms: Scientific Prospecting (2001–2021) and Systematic Review

Bruno Fonsêca Feitosa [1,*], Charlene Maria de Alcântara [2], Amanda Beatriz Sales de Lima [3], Adriano Sant'Ana Silva [2], Alfredina dos Santos Araújo [2], Mônica Tejo Cavalcanti [4], Edna Mori [5], Isaac Moura Araújo [6], Pablo Antonio Maia de Farias [6], Polrat Wilairatana [7,*] and Henrique Douglas Melo Coutinho [6,*]

[1] Faculty of Food Engineering, State University of Campinas, Monteiro Lobato, 80, University City "Zeferino Vaz", Campinas 13083-862, SP, Brazil
[2] Academic Unit of Food Technology, Federal University of Campina Grande, Jairo Vieira Feitosa, 1770, Pereiros, Pombal 58840-000, PB, Brazil
[3] Department of Rural and Animal Technology, State University of Southwest Bahia, Praça Primavera, 40, Primavera, Itapetinga 45700-000, BA, Brazil
[4] National Institute of the Semiarid Region, Francisco Lopes de Almeida, Serrotão, Campina Grande 58434-700, PB, Brazil
[5] CECAPE College. Av. Padre Cícero, 3917, Juazeiro do Norte 63024-015, CE, Brazil
[6] Department of Biological Chemistry, Regional University of Cariri, Av. Cel. Antonio Luiz, 1161, Crato 63105-000, CE, Brazil
[7] Department of Clinical Tropical Medicine, Faculty of Tropical Medicine, Mahidol University, Bangkok 10400, Thailand
* Correspondence: brunofonsecafeitosa@live.com (B.F.F.); polrat.wil@mahidol.ac.th (P.W.); hdmcoutinho@gmail.com (H.D.M.C.)

Table S1. Results of the keyword searches for articles in Portuguese (PT) and English (EN).

Keywords			Database			
			CAPES		SciELO	
Term 1	BO	Term 2	PT	EN	PT	EN
bioactiv*	AND	antib*	285	137,442	46	177
		antimicr*	298	65,407	56	203
		bactericidal	19	11,440	9	21
		biocidal	4	1,122	1	2
		"chemical agent"	1	427	-	18
phenol*	AND	antib*	3,168	139,073	130	224
		antimicr*	2,033	62,916	130	228
		bactericidal	79	11,001	23	37
		biocidal	11	1,770	-	4
		"chemical agent"	10	689	2	-
phytochemical	AND	antib*	162	21,425	33	95
		antimicr*	172	17,984	39	117
		bactericidal	9	2,802	2	15
		biocidal	4	155	-	-
		"chemical agent"	-	88	-	2
"essential oil" OR extract	AND	antib*	888	266,936	322	742
		antimicr*	1,000	104,795	390	822
		bactericidal	83	18,233	65	134
		biocidal	15	2,296	6	6
		"chemical agent"	5	908	2	4
Total			8,246	866,909	1,256	2,853

BO, Boolean operator; -, no article retrieved.

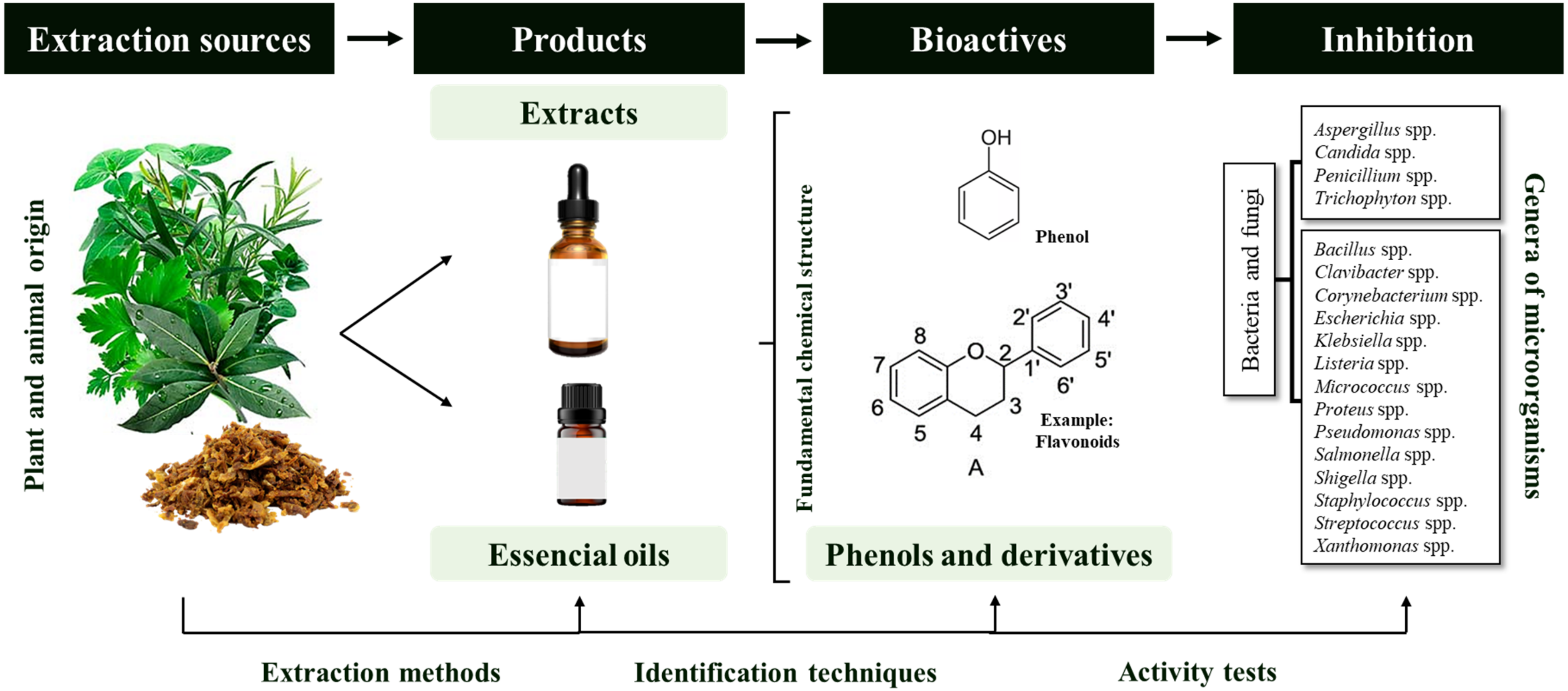

Figure S1. Graphical abstract of scientific information on bioactive natural products for microbial control.

Table S2. List of keywords used to search for scientific articles on bioactive natural products for microbial control in Portuguese and English.

**Database**	**Searches (27–30 April 2021)**	
	Portuguese	English
CAPES	bioativ* AND antib*; bioativ* AND antimicr*; bioativ* AND bactericida; bioativ* AND biocida; bioativ* AND "agente químico"; fenol* AND antib*; fenol* AND antimicr*; fenol* AND bactericida; fenol* AND biocida; fenol* AND "agente químico"; fitoquímico AND antib*; fitoquímico AND antimicr*; fitoquímico AND bactericida; fitoquímico AND biocida; fitoquímico AND "agente químico"; ("óleo essencial" OR extrato) AND antib*; ("óleo essencial" OR extrato) AND antimicr*; ("óleo essencial" OR extrato) AND bactericida; ("óleo essencial" OR extrato) AND biocida; ("óleo essencial" OR extrato) AND "agente químico"	bioactiv* AND antib*; bioactiv* AND antimicr*; bioactiv* AND bactericidal; bioactiv* AND biocidal; bioactiv* AND "chemical agent"; phenol* AND antib*; phenol* AND antimicr*; phenol* AND bactericidal; phenol* AND biocidal; phenol* AND "chemical agent"; phytochemical AND antib*; phytochemical AND antimicr*; phytochemical AND bactericidal; phytochemical AND biocidal; phytochemical AND "chemical agent"; ("essential oil" OR extract) AND antib*; ("essential oil" OR extract) AND antimicr*; ("essential oil" OR extract) AND bactericidal; ("essential oil" OR extract) AND biocidal; ("essential oil" OR extract) AND "chemical agent"
**Database**	Searches (1–4 May 2021)	
SciELO	bioativ* AND antib*; bioativ* AND antimicr*; bioativ* AND bactericida; bioativ* AND biocida; bioativ* AND "agente químico"; *fenol* AND antib*; *fenol* AND antimicr*; *fenol* AND bactericida; *fenol* AND biocida; *fenol* AND "agente químico"; fitoquímico AND antib*; fitoquímico AND antimicr*; fitoquímico AND bactericida; fitoquímico AND biocida; fitoquímico AND "agente químico"; ("óleo essencial" OR extrato) AND antib*; ("óleo essencial" OR extrato) AND antimicr*; ("óleo essencial" OR extrato) AND bactericida; ("óleo essencial" OR extrato) AND biocida; ("óleo essencial" OR extrato) AND "agente químico"	bioactiv* AND antib*; bioactiv* AND antimicr*; bioactiv* AND bactericidal; bioactiv* AND biocidal; bioactiv* AND "chemical agent"; *phenol* AND antib*; *phenol* AND antimicr*; *phenol* AND bactericidal; *phenol* AND biocidal; *phenol* AND "chemical agent"; phytochemical AND antib*; phytochemical AND antimicr*; phytochemical AND bactericidal; phytochemical AND biocidal; phytochemical AND "chemical agent"; ("essential oil" OR extract) AND antib*; ("essential oil" OR extract) AND antimicr*; ("essential oil" OR extract) AND bactericidal; ("essential oil" OR extract) AND biocidal; ("essential oil" OR extract) AND "chemical agent"
